# ICTs and the challenge of health system transition in low and middle-income countries

**DOI:** 10.1186/s12992-017-0276-y

**Published:** 2017-08-07

**Authors:** Gerald Bloom, Evangelia Berdou, Hilary Standing, Zhilei Guo, Alain Labrique

**Affiliations:** 1 Institute of Development Studies, University of Sussex, Brighton, BN1 9RE UK; 2 Cathay Capital Private Equity, Paris, France; 3 Johns Hopkins School of Public Health, Baltimore, USA; 4 Independent consultant, Brighton, UK

**Keywords:** E-Health, Digital health, Health knowledge economy, Universal health coverage

## Abstract

The aim of this paper is to contribute to debates about how governments and other stakeholders can influence the application of ICTs to increase access to safe, effective and affordable treatment of common illnesses, especially by the poor. First, it argues that the health sector is best conceptualized as a ‘knowledge economy’. This supports a broadened view of health service provision that includes formal and informal arrangements for the provision of medical advice and drugs. This is particularly important in countries with a pluralistic health system, with relatively underdeveloped institutional arrangements. It then argues that reframing the health sector as a knowledge economy allows us to circumvent the blind spots associated with donor-driven ICT-interventions and consider more broadly the forces that are driving e-health innovations. It draws on small case studies in Bangladesh and China to illustrate new types of organization and new kinds of relationship between organizations that are emerging. It argues that several factors have impeded the rapid diffusion of ICT innovations at scale including: the limited capacity of innovations to meet health service needs, the time it takes to build new kinds of partnership between public and private actors and participants in the health and communications sectors and the lack of a supportive regulatory environment. It emphasises the need to understand the political economy of the digital health knowledge economy and the new regulatory challenges likely to emerge. It concludes that governments will need to play a more active role to facilitate the diffusion of beneficial ICT innovations at scale and ensure that the overall pattern of health system development meets the needs of the population, including the poor.

## Background

The rapid pace of mobile phone adoption, with its promise of universal connectivity, lends credence to beliefs that the latest generation of information and communication technologies (ICTs) will support substantial beneficial changes in the organisation of the health sector [1–3]. This has led donor agencies and foundations to invest in many digital health interventions in low and middle-income countries, in the hope that they will provide a way to address major deficiencies in access to safe, effective and affordable health services, especially by the poor [4]. It has also stimulated large private sector investments in pursuit of niches in rapidly changing markets.

The aim of this paper is to contribute to debates about how governments and other stakeholders can influence the application of ICTs to increase access to safe, effective and affordable treatment of common illnesses, especially by the poor. It draws on the work of a number of analysts, who have shown how the introduction of a new technology can disrupt the way a sector is organised and, eventually, lead to a transition to a different arrangement with new companies, new kinds of partnership and new regulatory arrangements [1, 5, 6]. First, it argues that a health sector is best conceptualized as a ‘knowledge economy’. This supports a broadened view of health service provision that includes formal and informal arrangements for the provision of medical advice and drugs. This is particularly important in countries with a pluralistic health system, with relatively underdeveloped institutional arrangements. It then argues that reframing the health sector as a knowledge economy allows us to circumvent the blind spots associated with donor-driven ICT-interventions and consider more broadly the forces that are driving e-health innovations.

The subsequent section presents the findings of studies of digital health in Bangladesh and China, which explore how the information and health sectors are responding to the changing technological possibilities. These are small scale studies of different aspects of digital health in each country. They offer an opportunity to explore how information and health sector innovations are opening up possibilities for reconfiguring the delivery of outpatient health advice and treatment. These explorations also raise fundamental questions about regulation and governance to protect the interests of the poor and ensure that health information remains at the service of public health. The paper concludes with a discussion of the role of governments in supporting and influencing the direction of development of ICT-enabled health systems.

## Knowledge asymmetries and the health sector

We view the health sector as a knowledge economy, which makes widely available the benefits of expert medical knowledge and specialised commodities, such as pharmaceuticals [7]. Although many spheres of economic activity involve asymmetries of knowledge and expertise between providers and consumers, the health knowledge economy has unique characteristics because of the potentially deleterious consequences of an ill-informed response to a health challenge for an individual and/or a community. This bestows power on possessors of health-related expertise and a risk that they will abuse this power [8, 9]. To redress this imbalance, societies have evolved institutional arrangements to ensure that experts are competent and limit the degree to which certified experts can use their power for personal or corporate gain. These arrangements include self-regulating professions, watchdog organisations, and professional regulatory bodies. The state also commonly funds research, training of medical experts and provision of some health services. The existence of extra-market arrangements for protecting the public good is a characteristic of an effective health sector. The way these arrangements function is strongly influenced by the interests and understandings of users, providers, gatekeepers and by the context of broader configurations of power and expertise. This understanding of a health knowledge economy shifts analysis away from the impact of individual digital health interventions towards an examination of the way that new technologies and the actors behind them are beginning to disrupt these configurations both globally and locally and the potential consequences for large-scale change.

This paper focuses on low- and middle-income countries with a pluralistic health sector, where governance and institutional arrangements are much less entrenched than in the advanced market economies [7, 10]. During the twentieth century the latter countries experienced significant political debate about the organisation of their health sectors. They created institutional arrangements to protect their population against major health shocks and provide access to safe, competent and trustworthy care. These arrangements represent political agreements that have become deeply embedded in their society and are, therefore, difficult to change [11–13]. Some analysts have argued that the relative newness of the so-called “emerging markets”, especially around the telecommunications and health sectors, mean that they face fewer constraints to the emergence and rapid spread of new kinds of organisation supported by ICTs [14, 15].

## From digital health interventions to the evolution of health knowledge economies

Both the use of mobile phones and access to the internet have increased rapidly over the past decade in low- and middle-income countries.^1^ This has stimulated many local applications of information technology in the management of health facilities and programmes, the provision of information and advice to health workers and patients and the strengthening of links between front-line health services and more highly qualified doctors [16]. However, a recent study, which included interviews with key informants in the digital health community, reported that very few could identify many examples of digitally-enabled health service innovations that successfully went to scale [17]. Systematic reviews of different types of ICT-supported health intervention in low- and middle-income countries, funded by donors or foundations, have all reached similar conclusions about the outcomes of these interventions [18–23]. The small number of available evaluations has mostly shown the feasibility of interventions that address a narrowly defined health issue, such as improving the coverage of ante-natal services or the timeliness of routine immunizations. A small number have demonstrated improvements in decision-making by health workers or the general public. Even fewer have demonstrated impact on health services and/or health outcomes at scale [21]. This is partly because of a lack of good quality studies, but it also reflects the complex process through which a series of technological innovations can ultimately lead to major changes in a sector’s organisation. Expectations invested in relatively modest pilot interventions would seem to have been unrealistic.

Analysts have argued that evaluations of ICT-enabled health interventions need to shift from testing whether an innovation has a direct effect on health outcomes to exploring the wider impact of ICT solutions on alleviating health system constraints and improving performance [16]. A recent paper follows the World Health Organization (WHO) in defining a health system as “all activities whose primary purpose is to promote, restore or maintain health” and identifies several functions that digital health interventions have addressed [4]. It argues that health systems can incorporate applications that address critical constraints that impair their performance. Although this approach offers insights into the possibilities afforded by digital health, its focus on the normative and technical components of a health system doesn’t adequately consider two important aspects of a health knowledge economy: the sets of social, economic and political interests which shape processes and outcomes; and the large array of informal and external relationships, commodities and services, which provide health-related information, advice or treatments.

The way an analyst frames a system and the boundary he or she draws around its constituent parts influences both the topics of analysis and the policy options considered [24]. Using a health knowledge economy lens means that a more inclusive boundary can be drawn. It frames the health system to include all actors that provide knowledge and advice or specialised medical commodities to the population and takes into account user and market driven ICT practices. This has two consequences. First, it includes individuals and organisations within and outside the system of formal provision. This is important in a pluralistic health system, where many people seek advice and buy pharmaceuticals from drug stores, or service providers working outside the regulatory framework [7, 25]. Second, it draws attention to the increasing involvement in the health sector of information and communications organisations such as mobile phone operators, local ICT entrepreneurs, advertising agencies and large knowledge companies.

Technological developments are enabling the emergence of new types of organisation and new forms of partnership and network that cross the boundaries between the health, knowledge and communications sectors, stimulated by several demand and supply-side factors [1, 14, 26]. More people are living with chronic conditions without much support from the formal health system, especially in low- and middle-income countries [2, 27, 28]. The development of evidence-based treatment guidelines that link symptoms, diagnostic tests and basic treatment may be stimulating a shift from “medical dominance” to “managed consumerism” with people taking more responsibility for their own “health maintenance” in the advanced market economies [29]. Also, new forms of corporate organisation of health service delivery have become increasingly prominent [30–32]. The development of communications media, especially mobile phones, is creating a way to provide low-cost access to expert knowledge and advice, and the development of mobile phone payment mechanisms is making innovative business models easier to implement.

According to Schmit and Cohen [33], the explosive increase in access to mobile phones and the internet by relatively poor people in low- and middle-income countries is creating new business opportunities and generating new regulatory challenges. In many of these countries, a high proportion of economic transactions takes place outside a formal regulatory framework [34]. Meanwhile, large corporations from a number of countries, including China and India, are establishing a global presence. A recent report by Dobbs et al. [15] argues that these countries are becoming sources of disruption of global markets because of the rapidly rising demand for services associated with economic growth and urbanisation and their less entrenched institutional arrangements. Despite these possibilities, recent reviews have shown that progress by private service delivery organizations in meeting health-related demands has been limited [35] and the spread of digital health services has been modest [3]. The following section presents information from Bangladesh and China, which shows that new types of organisation and partnership are emerging, which may eventually create the possibility of change at scale.

## Bangladesh and China: New intermediaries, new dynamics?

Bangladesh and China have pluralistic health systems with big differences between urban and rural areas in the availability of good quality and trusted health facilities. Both countries have high levels of mobile phone coverage, a dynamic ICT sector and governments that are encouraging the development of this sector. There are also significant differences. China has experienced sustained economic growth over many years leading to a dramatic fall in poverty and rapid increases in disposable income. It is implementing a series of reforms aimed at strengthening government health services. It has large and well-established companies in both the pharmaceutical and information sectors, which are increasingly engaged in global markets. Bangladesh is typical of a number of low-income countries, with a much smaller internal market and very few companies with a global reach, although it has a well-established national pharmaceutical industry, a basic government health service infrastructure and major health service delivery NGOs. It also has growing capacity in software development and applications. Its e-health sector largely depends on international donors and foundations for investment finance [36].

### Digital health innovations in the Bangladesh health sector

The data on Bangladesh are derived from a mapping and review of 26 e-health interventions in 2012 [37], a series of in-depth interviews with e-health innovators in Dhaka as part of a broader study of innovations and health system change in Bangladesh [36] and a survey of 800 households in rural and urban localities to study health information seeking behaviour and weighted towards lower income households [38]. These studies describe a variety of actors and emergent partnerships and networks. The Ministry of Health has established an e-health administrative unit, which is integrating ICTs into planning and management and establishing a telephone medical advice line. Several start-up companies have developed ICT applications and different knowledge intermediaries provide information on health issues on a website or in SMS messages. These intermediaries include large NGOs, research institutes, social businesses and private entrepreneurs based within and outside Bangladesh. These initiatives are mostly funded by grants from donor agencies or foundations. The study found several innovative partnerships and networks. One m-health company had linked to local (untrained) village doctors/drug sellers to offer a package of basic services. Another had partnered with a very large service delivery NGO and a national retail chain to create a website on maternal and child health and send SMS messages to pregnant women. Another national retail chain had established a health website and advice line, linked to an online shop. Several mobile phone operators had launched health advice lines. The study found that company leaders were enthusiastically seeking a niche in what they perceived to be a potentially large market in the future. However, the only ones that were financially secure were linked to an established retail chain or mobile phone operator.

The household survey found that only a tiny proportion of people had actually used their mobile phones to seek health information and there was little awareness of websites with health information or SMS health messages [38]. The situation was different for college students, a significant proportion of whom used mobile phones to seek information on sexual health or physical appearance, relying especially on Facebook as a source of information and advice [39]. There were significant differences in the way that men and women used phones to access information, reflecting underlying gender relationships and power dynamics within households. In particular, women’s access to phones was often mediated by family gatekeepers such as husbands and fathers who controlled both the finance and the social interactions that wives and daughters were allowed.

Overall, there was a lot of innovative activity in Bangladesh, mostly financed by non-commercial innovation grants and internal investments by established companies. Some promising partnerships were emerging and several companies were developing an online presence, but there had been little substantial impact on the way that most people manage their health problems.

### Private investment in digital health in China

The information on China comes from a scoping study of the digital health sector by (Guo Z. Implication of mHealth Development and Application to the China Health Reform, unpublished). This consisted of a review of published data, online news and information sources and the websites of companies involved in the sector. The study found a very sharp increase in e-health investments, reaching 15.75 billion RMB ($2.5 billion) in 2015. The turnover is projected to reach 2.5 billion rmb a year by 2017. One major area of investment is in the online supply of pharmaceuticals. This has been stimulated by government efforts to reduce the incentives for health facilities to encourage excessive use of drugs by ending their ability to earn a profit from supplying these products [40]. Organizations developing these online services include pharmaceutical manufacturers, pharmaceutical retail chains and very large online shopping platforms. At least one of these shopping platforms has acquired a retail pharmacy chain. Chinese law defines a category of drugs that can only be supplied on a doctor’s prescription, which some online platforms have circumvented by employing doctors, or ignoring the law [41]. Companies are also lobbying for a change in the regulatory framework to permit electronic prescriptions. This would facilitate the creation of new types of relationship between health facilities and pharmaceutical suppliers. An article in the China Daily [42], posted on the website of the State Council, indicated that government is considering this kind of change. However, there has been little movement since then, suggesting that the decision is contested. The ultimate decision will strongly influence future developments, since it could open access to a very large market.

A second area of business is the provision of online medical advice services. One example is Spring Rain Doctor, which provides online consultations with a doctor. It is also creating an algorithm-driven electronic advice service. In 2015 it claimed to have 45 million users who posted about 60,000 medical queries a day [43]. A third area of business involves the use of devices to monitor health metrics and enable people to manage their fitness or their control of a health problem. One example is a platform to support people with diabetes, which includes the use of a device to monitor blood sugar.

The rise in investment in digital health is so recent that it is difficult to know whether it reflects the opening up of big business opportunities or a short-term bubble, with inflated statements of investment commitments. The number of companies involved and the size of proposed investments suggest the former. The government has stated its support for e-health, but many issues are being negotiated. These include the legalisation of electronic prescriptions, the establishment of an enforceable framework to regulate the quality of diagnostic devices and of the advice provided and the development of links between digital health services and government health facilities. The size of China’s health care market is big and if digital health services become firmly established, Chinese firms would have great possibilities for taking these services abroad.

Bangladesh and China illustrate the degree to which new actors are becoming involved in the health knowledge economy. In Bangladesh, mobile phone operators, retail chains selling health-related commodities and large service delivery NGOs have built partnerships with small tech start-up companies. Facebook has become an important source of health information amongst the young. In China, large pharmaceutical manufacturers and retailers and internet platforms are playing a leading role. There are also a large number of tech start-ups. There is evidence of new kinds of partnership that link organisations that (i) provide expert advice using trained professionals and/or treatment guideline algorithms, (ii) give access to low cost diagnostic technologies, such as testing for blood sugar, and (iii) have the capacity to supply drugs and health-related commodities. Despite the many developments described above, the impact of ICTs on the organisation and performance of health knowledge economy of these countries has, to date, been limited.

## Discussion

### Are ICTs disrupting the health knowledge economy?

A senior official of a donor agency recently asked one of the authors whether their agency should take digital health into account in the provision of support for health system strengthening. There is no simple answer to this question. As discussed above, most donor-funded investments in digital health have not yielded big health benefits. However, the studies in Bangladesh and China found evidence of a variety of emergent organisations, inter-organizational relationships and business models, suggesting the possibility of future changes at scale. Elsewhere too, the growing tendency of large American internet companies to offer health-related services, in the form of fitness-related devices, systems to support people with chronic illnesses and so forth, indicates their recognition of potentially big business opportunities.

We suggest that the relatively slow development of digital health reflects the following special characteristics of the health knowledge economy. First, a useful health service in a country like China or Bangladesh needs to include a combination of diagnosis, expert advice and drugs. This is likely to involve relationships between organisations with different roles, responsibilities and values in the health and communication sectors. Second, health systems often combine public funding of some services with out-of-pocket payment for others, so that business models need to combine funding from multiple sources. Third, well-functioning health systems must be embedded in institutional arrangements for accountability and efforts to reshape markets and associated regulatory frameworks are complex and politically charged. The need to achieve progress on each of the above, has reduced the speed with which new technological possibilities have been translated into large-scale changes in the way health services are organised [1].

Debates about the desirability of further investment in digital health reflect the different time horizons and attitudes to risk of large ICT companies, venture capital funds, donor agencies and governments. Some large companies take a long view aimed at creating a niche in a rapidly changing economy and are not afraid to incur significant losses in the short term. For example, Google invested heavily in creating detailed city maps, to produce a digital infrastructure. Other companies have experienced losses, whilst building an enormous customer base, which eventually generated profits. A number of companies are investing to establish future positions in the health knowledge economy. Donor agencies and governments, on the other hand, have a shorter time horizon, preferring low-risk investments likely to yield immediate benefits. Recent efforts to coordinate donor investments in digital health reflect the beginnings of a strategic shift, with mechanisms such as the Health Data Collaborative (HDC) and USAID’s Digital Health Initiative emerging to support country-level capacity development [44]. However, these initiatives have largely focused on public sector developments. We argue that governments and donor agencies need to adopt a longer-term perspective in their interventions in the health knowledge economy to support developments in the public and private sectors that build local capacity and meet the needs of the poor [45].

### Regulatory challenges in the health knowledge economy

The developments described above pose big regulatory challenges and governments need to modify and strengthen institutional arrangements to address them. This will involve changes to regulations that block the development of potentially beneficial services. For example, there may be rules that reserve the right to provide advice on the use of many drugs to a licensed doctor, even though large numbers of people buy these products from local drug sellers without a prescription. It will also involve additions to the regulatory framework.

One challenge arises from the increasing use of treatment guidelines. This builds on previous investments by the governments of several advanced market economies to support the generation and synthesis of knowledge to inform evidence-based medical care. This knowledge has been incorporated into diagnosis and treatment guidelines in text books and manuals used by providers of medical care and increasingly by the general public. The use of guidelines for a growing number of treatment decisions is diminishing the role of clinical judgement in the management of many common health problems [29]. This is likely to reduce the capacity of doctors to control the use of drugs for these conditions. Other approaches will be needed to encourage people to use them appropriately.

The translation of treatment guidelines into computerized decision-making algorithms is increasing their influence. Algorithms that provide advice on the basis of answers to simple questions and diagnostic information, such as blood pressure, temperature and blood sugar can substantially increase the ability of people to make use of treatment guidelines. Their incorporation into easy-to-use smartphone apps will further simplify their use.

The producers of algorithms can influence the decisions of many people. Their underlying assumptions, cultural understandings and financial interests are likely to affect the content of the algorithms [46]. For example, if the questions an algorithm asks and the data it uses focus on the relationship between pharmaceutical treatment and health, it will provide advice on the choice and dosage of drugs, but not on other factors that influence health, such as diet, lifestyle and exposure to environmental toxins. This could lead to an excessive reliance on drugs to control risk factors for non-communicable diseases. Another example is the establishment of a new diagnosis. This could have major financial implications if it were to justify a particular treatment regime [47, 48]. In some cases, pharmaceutical companies have attempted to influence the definition of a diagnosis as a strategy for creating markets for their products [49]. This applies especially to the growing number of “lifestyle”-related uses of pharmaceuticals for altering moods, increasing libido, building or losing weight, increasing athletic performance and so forth. The boundary between “lifestyle” and “medical” decision-making algorithms is becoming increasingly difficult to define in the face of the rising burden of chronic non-communicable diseases, including mental illness, and of measures to reduce symptoms and control risk-factors, such as hypertension.

The public domain has tended to lead the development of treatment guidelines, with a substantial involvement of organised medical professions and training and research institutions. The character of many digital health applications, in contrast, is largely opaque. A study of the top-rated medical and health applications available through Apple’s App store reveals that most make unsubstantiated claims to medical authority, leaving both expert and lay users in the dark about where the information and advice they provide is derived from and whether the producers of the applications have links with companies that sell pharmaceuticals or diagnostic equipment [50, 51].

Two developments are likely to increase the importance of health-related algorithms and the governance challenges associated with them. The first concerns the increasing availability of low-cost diagnostic technologies in the form of smartphone and computer attachments or standalone devices meant to be used by patients, front line medical staff or other suppliers of drugs and health-related commodities. The second concerns automated processes for updating the content of original algorithms on the basis of incoming data (machine learning). The continuous collection of data that links indicators, such as blood pressure and blood sugar to specific treatments creates the possibility of collecting a large body of data that could guide future treatment regimens. This raises issues regarding the accuracy of the data collected and also the specific “research” questions that drive the data collection. The organisations that own the data and use them to revise algorithms will accrue increasing influence.

The availability of health-related algorithms could become especially important in countries with pluralistic health systems, where people take a lot of responsibility for their own health care and access to trustworthy treatment guidelines could be particularly useful [28, 52]. However, inappropriate guidelines or apps aimed at generating new markets for pharmaceuticals or diagnostic equipment pose a particular risk in these countries due to poor regulation and lack of consumer protection. Lewis and Wyatt [53] present a framework that uses a combination of usage scenarios, contextual factors and app complexity to assess the risk of harm from a so-called health app.

There are a variety of potential regulatory approaches for addressing these issues. Some health problems may be relatively minor and regulators could follow a strategy of “buyer beware” by strengthening consumer protection and focusing regulations on informing the public about the contents of any products and preventing false and misleading claims. In other cases, major deleterious consequences could arise from the provision of misinformation and inappropriate treatment, making information asymmetry an important consideration. Governments and organised professional and expert bodies have an important role to play in these cases. This may involve the development of treatment guidelines and algorithms as national or global public goods to be made available for use by both public and private health service providers.

Alternatively, it may be appropriate to regulate the production and use of treatment algorithms. Mechanisms are needed to ensure that the organised professions and the medical and nursing schools associated with them, pharmaceutical companies and newer entrants in the health knowledge economy are accountable for the advice they provide. This will require strong strategic leadership by national governments and international organisations.

In China and Bangladesh, the links between providers of medical advice and treatment and suppliers of pharmaceuticals are complex. In Bangladesh, informal village doctors and drug sellers, working outside the regulatory framework, are important sources of both advice and treatment, especially for the poor. In that country, there have been several attempts to link a digital health company with village doctors, as a means of improving the practice of the latter. In China, where the formal retail sector developed rapidly since the beginning of the transition to a market economy in the 1980s, several online shopping companies are now making large investments to establish links with local shops and create a capacity to make next-day deliveries. These examples illustrate how companies can use information technologies to enable people to link to a network of small businesses, which can supply goods and services. This kind of network that links local suppliers of pharmaceuticals to a source of treatment algorithms and a means for monitoring drug quality could provide a low-cost way to meet health care needs of the relatively poor. It might involve an internet platform and pharmaceutical wholesalers and retailers. But, there are governance risks concerning the management of conflicts of interest between those of clients, who are seeking evidence-based and cost-effective ways to deal with health problems, and businesses, whose revenues depend on the volume of sales of pharmaceuticals and diagnostic devices. Governments will need to play new and challenging stewardship roles as these changes go to scale.

### Strengthening public health in the interests of the poor: Supporting transitions in the health knowledge economy

The Bangladesh and China cases illustrate the different kinds of organisation that are becoming involved in the health knowledge economy and the variety of new kinds of partnership that are being established. It is possible to envisage quite different pathways of development as new possibilities for organising access to health services have impact at scale. Some pathways could result in big increases in access to appropriate advice and effective treatment. Others, however, could reflect the interests of powerful stakeholders, such as the producers and distributors of diagnostic tests and drugs, and encourage unnecessary use of these products. The “choice” of development pathways will be strongly influenced by political factors and the actions of government and other stakeholders to establish new kinds of partnership and reform the regulatory framework.

Developments in the broader knowledge economy are likely to influence the health knowledge economy. In the past 20 years a number of information companies have grown very quickly to take advantage of short-term monopoly positions, becoming very large corporations that combine provision of access to the internet, production of knowledge content and the diffusion of content through the mass media [54, 55]. Other companies like Google, Facebook, Amazon and Alibaba have become actors in political negotiations about the shape of knowledge industry markets [56–58]. These companies are seeking niches in the health knowledge economy and will eventually establish relationships with organisations that deliver health services and supply health-related commodities. This opens up the possibility of the emergence new types of powerful organisation that seek to influence the regulatory framework.

In China, pharmaceutical producers, retail pharmacy chains and large online shopping platforms are making substantial investments to develop digital health services. They are engaging with the organised health system and health regulatory agencies to influence the institutional arrangements. In Bangladesh, retail chains are building their presence in a market place that is changing more slowly, certainly in terms of consumer demand. Much of the investment in Bangladesh has been in the form of grants by donor agencies to relatively small ICT companies and by mobile phone operators seeking added-value lines of business. As Bangladesh’s consumer base increases with economic growth, these increased market opportunities are likely to attract larger players in the knowledge industry markets. One particularly important trend is the increasing role of digital platforms as intermediaries between different stakeholders. In China and Bangladesh, health sector intermediaries include websites of existing brick-and-mortar suppliers of drugs and health-related products, telemedicine platforms and online platforms that offer a wide variety of goods and services. These intermediaries are likely to gain influence as gatekeepers to information and advice.

## Conclusions

Christensen et al. [1] argue that a major health system transition entails the creation of a new “value network”. By that they mean organisational structures and regulatory arrangements that make possible the rapid growth of new types of service delivery organisation, the creation of new kinds of relationship between health sector organisations and between them and health financing agencies. Wilson et al. [26] make a similar argument in advocating that investments in digital health should define “institutionalization” as an appropriate goal. They suggest that all stakeholders involved in the ICT-enabled health sector in low- and middle-income countries need to build a common vision for achieving this goal and that governments and funding agencies should support investments to achieve the vision. We agree with these reflections but suggest that more attention should be paid to the influence of conflicts of interest and unequal power relationships on the direction of change. Castells [55] emphasises the influence of the coordinating and regulatory functions of the state and argues that the networks of power constructed around the state play a fundamental role in the overall pattern of development of the knowledge economy. This includes the efforts by some stakeholders to increase their competitive advantage by influencing regulatory arrangements in ways that could conflict with the interests of the public [5]. For example, they could oppose regulations meant to ensure that providers of medical advice do not have a financial interest in promoting high levels of drug use.

Governments already play an important role in most countries’ health sector. They will need to play an active role in shaping health knowledge economy markets and ensuring that the interests of citizens, particularly the poor and politically weak, are represented. Otherwise there is a risk that new value networks will respond largely to the interests of the better off and of the suppliers of health-related goods. There is a need for more work on mapping the participants in the health knowledge economy and their strategies for building market share to inform debates about regulatory reform.

Governments, donor agencies and foundations need to reassess their strategy for supporting digital innovation in the health sector to take the above considerations into account. There is still a need for investment in innovations that address specific health and health service needs. However, this needs to be complemented by strategic investments in the establishment of partnerships and networks that have the capacity to use innovative approaches to meet the health needs of poor clients at scale. Other measures are needed to strengthen the capacity of government and other stakeholders to play an effective regulatory and stewardship role. This may include investment in the creation of algorithms for treatment of common health problems and the supply of them to potential health intermediaries, regulation of cross-sector ownership to reduce the incentives to oversupply drugs or diagnostic tests and new kinds of national and global regulation of the provision of medical advice. The lessons from other sectors suggest that once a tipping point is reached, the process of change can be very rapid. Governments need to engage actively in the innovation process, assess potential risks and set in place strategies to mitigate these risks as digital innovations increasingly disrupt health knowledge economies.

## Abbreviations

ICT: Information and communication technology
NGO: Non government organization
WHO: World Health Organization

## Footnotes

1
http://www.itu.int.en.mediacentre/Pages/2016-PR30.aspx
